# A Simplified Method for Generating Purkinje Cells from Human-Induced Pluripotent Stem Cells

**DOI:** 10.1007/s12311-017-0913-2

**Published:** 2018-02-03

**Authors:** Lauren M. Watson, Maggie M. K. Wong, Jane Vowles, Sally A. Cowley, Esther B. E. Becker

**Affiliations:** 1 Department of Physiology, Anatomy and Genetics, University of Oxford, Oxford, UK; 2 Sir William Dunn School of Pathology, University of Oxford, Oxford, UK

**Keywords:** Stem cells, Cerebellum, Purkinje cells, Ataxia, Neurodegeneration, Development

## Abstract

The establishment of a reliable model for the study of Purkinje cells in vitro is of particular importance, given their central role in cerebellar function and pathology. Recent advances in induced pluripotent stem cell (iPSC) technology offer the opportunity to generate multiple neuronal subtypes for study in vitro. However, to date, only a handful of studies have generated Purkinje cells from human pluripotent stem cells, with most of these protocols proving challenging to reproduce. Here, we describe a simplified method for the reproducible generation of Purkinje cells from human iPSCs. After 21 days of treatment with factors selected to mimic the self-inductive properties of the isthmic organiser—insulin, fibroblast growth factor 2 (FGF2), and the transforming growth factor β (TGFβ)-receptor blocker SB431542—hiPSCs could be induced to form En1-positive cerebellar progenitors at efficiencies of up to 90%. By day 35 of differentiation, subpopulations of cells representative of the two cerebellar germinal zones, the rhombic lip (Atoh1-positive) and ventricular zone (Ptf1a-positive), could be identified, with the latter giving rise to cells positive for Purkinje cell progenitor-specific markers, including Lhx5, Kirrel2, Olig2 and Skor2. Further maturation was observed following dissociation and co-culture of these cerebellar progenitors with mouse cerebellar cells, with 10% of human cells staining positive for the Purkinje cell marker calbindin by day 70 of differentiation. This protocol, which incorporates modifications designed to enhance cell survival and maturation and improve the ease of handling, should serve to make existing models more accessible, in order to enable future advances in the field.

**Electronic supplementary material:**

The online version of this article (10.1007/s12311-017-0913-2) contains supplementary material, which is available to authorized users.

## Introduction

The role of the cerebellum in the coordination of smooth movements is well established [1]. More recently, multiple lines of evidence have implicated this critical brain region in perception, emotion and cognition, via its extensive connections with cortical and subcortical centres [2]. It therefore follows that cerebellar dysfunction results not only in motor deficits but also to non-motor symptoms in more complex neurological conditions [3]. Indeed, impairment of the cerebellar circuitry has been linked to the development of numerous motor and non-motor diseases, including ataxia, dystonia and Huntington’s disease, as well as autism spectrum disorders and schizophrenia.

Despite its apparent regularity, the development of cerebellar circuitry is remarkably complex, involving two germinal zones—the rhombic lip (responsible for the generation of cerebellar glutamatergic neurons including granule cells, unipolar brush cells and projection neurons of the deep cerebellar nuclei) and the ventricular zone (which gives rise to cerebellar GABAergic neurons, including Purkinje, Lugaro, Golgi, basket and stellate cells and Bergmann glia) [4, 5]. The result is an intricately ordered structure, containing more neurons than any other brain region. Central among these are the Purkinje cells, the sole output neurons of the cerebellar cortex, whose large size and elaborate dendritic arbour not only make them well suited to receiving and processing input from granule cell parallel fibres, but also render them particularly susceptible to the proteostatic insults and disturbances in ion channel function associated with cerebellar disease [6].

Dissecting the molecular mechanisms underlying this vulnerability requires a suitable model for the study of cerebellar development and degeneration. In the case of humans, brain tissue from affected individuals is difficult to obtain and is typically acquired postmortem, offering only limited insights into an advanced stage of pathology. Mouse models, on the other hand, can be studied throughout development, and are amenable to genetic manipulations required to mimic both Mendelian and complex diseases. Nevertheless, they are hampered by species-specific differences in brain structure and gene function, which may affect the interpretation of results.

Arguably the biggest breakthrough in the field of human neurodegenerative disease research to date has come in the form of induced pluripotent stem cells (iPSCs) [7]. These cells can be derived directly from patients carrying disease-causing mutations, or engineered to carry genetic defects of interest. Importantly, they also have the potential to differentiate into any cell type of the body, offering the unique opportunity to study human neurons in vitro without the need for invasive surgical techniques.

iPSC-based models have been used to shed light on the pathology of numerous neurodegenerative diseases, including several of the spinocerebellar ataxias, and Huntington’s disease (reviewed in [8]). However, studies of cerebellar neurons remain remarkably rare, largely due to the complexities of development and neuronal architecture mentioned above. To date, only a handful of studies have succeeded in generating Purkinje cells from human or mouse pluripotent stem cells [9]. The earliest of these attempted to mimic early cerebellar developmental signals through the stepwise delivery of bone morphogenic proteins, mitogens and neurotrophins throughout the differentiation process, with relatively low differentiation efficiencies [10, 11]. More recent approaches have therefore turned to the induction of endogenous signals mimicking cerebellar patterning, by adding a combination of fewer factors, designed to initiate endogenous early developmental signalling cascades [12, 13].

**Fig. 1:**
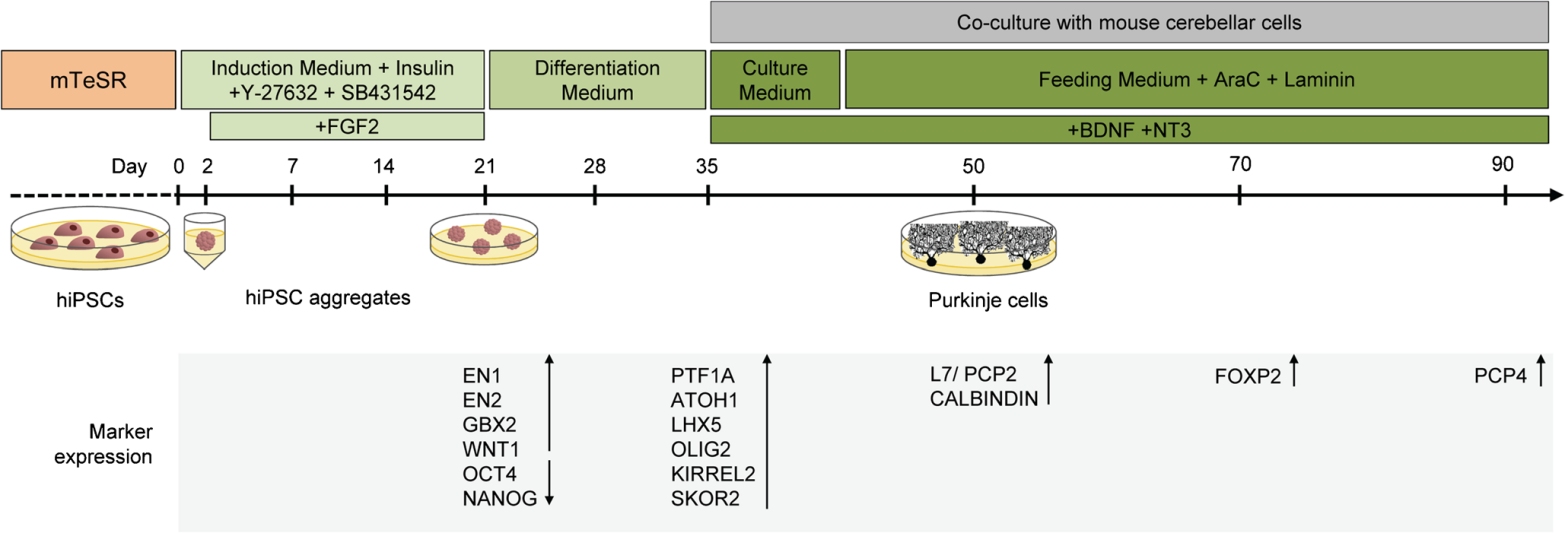
Overview of cerebellar neuronal differentiation protocol. hiPSCs were typically seeded at 12,000 cells/well in ultra-low attachment plates and cultured in suspension for 35 days before dissociation and co-culture with E18.5 cerebellar cells. Gene and protein expression was assessed at various time points by means of qPCR and immunofluorescence

Here, we report the successful adaptation of a method [13] for the differentiation of cerebellar Purkinje cells from human iPSCs (Fig. 1). Through multiple rounds of optimisation, we have defined the critical parameters necessary for robust, reproducible generation of these neurons in culture. In so doing, we aim to make this protocol more accessible for others wishing to employ such a model for the study of human cerebellar development and disease.

## Material and Methods

### Ethics

Ethics approval was obtained from the National Research Ethics Service Committee, South Central—Southampton B (REC reference number 12/SC/0106). Written informed consent was obtained from all participants, according to the Declaration of Helsinki (WMA 1997). All animal work was carried out in accordance with UK Home Office regulations.

### Reprogramming, Culture and Maintenance of iPSCs

Reprogramming of donor fibroblasts to iPSCs was performed as described in the Supplementary Methods. Three iPSC lines were used, representative of two unaffected control individuals: a 49-year-old male OX3 [14] (clones OX3-6 and OX3-7) and a 67-year-old female AH017 (clone AH017-3) [15]. Characterisation of the previously unpublished clone, OX3-7, is provided in Fig. S1. iPSCs were maintained in feeder-free conditions on hESC-qualified matrigel (Corning, Corning, NY, USA), in supplemented mTeSR (Stem Cell Technologies, Vancouver, Canada). Cells were passaged 1:3 every 2–5 days, using 0.5 mM EDTA (Invitrogen, Carlsbad, CA, USA) [16].

### Generation of Hindbrain Neural Progenitors from iPSCs

Reagents were purchased from Thermo Fisher Scientific, unless otherwise stated. On day 0, iPSCs (typically 1–3 wells per line, at approximately 75% confluence) were dissociated for 5 min at 37 °C in prewarmed TrypLE Express. TrypLE Express was then diluted 1:10 in prewarmed 1× Dulbecco’s phosphate-buffered saline (DPBS), and the cells were centrifuged for 5 min at 228*×g* (1000 rpm). Following centrifugation, the cell pellet was gently resuspended to give a single cell suspension, in induction medium, consisting of Iscove’s modified Dulbecco’s medium/Ham’s F12 1:1, insulin (7 μg/ml, Sigma-Aldrich, St. Louis, MO, USA), crystallisation-purified bovine serum albumin (BSA, 5 mg/ml, Sigma-Aldrich), chemically defined lipid concentrate (1%), monothioglycerol (450 μM, Sigma-Aldrich), apo-transferrin (15 μg/ml, Sigma-Aldrich) and penicillin/streptomycin (1%). At this stage, induction medium was additionally supplemented with 50 μM Y-27632 (Sigma-Aldrich) and 10 μM SB431542 (Tocris, Bristol, UK). To allow for reaggregation, 12,000 cells/well were transferred to three to four low-adhesion V-bottomed 96-well PrimeSurface culture plates (Sumitomo Bakelite, Tokyo, Japan) in this supplemented induction medium, and incubated for 48 h at 37 °C, 5% CO_2_. Recombinant fibroblast growth factor 2 (FGF2, 50 ng/ml, R&D Systems, Minneapolis, MN, USA) was added to the culture on day 2.

A one-third volume replacement was performed on day 7, using fresh induction medium without Y-27632 or 10 μM SB431542, and a further full volume medium replacement was performed on day 14.

On day 21, cell aggregates were transferred from 96-well plates to low-adhesion 24-well plates (Corning). A micropipette with a cut-off tip was used to transfer approximately four aggregates into each well, and aggregates were incubated for 14 days in differentiation medium, consisting of neurobasal medium supplemented with GlutaMAX (1%), N2 (1%) and penicillin/streptomycin (1%). A full-volume replacement with fresh differentiation medium was performed on day 28.

### Dissociation and Co-Culture of Purkinje Cells

On day 35 of suspension culture, cell aggregates were transferred into microcentrifuge tubes (approximately 10 aggregates per tube) using a micropipette with a cut-off tip. Aggregates were washed twice with HHGN, consisting of 1× HBSS supplemented with 2.5 mM HEPES pH 7.3–7.5, 35 mM glucose and 4 mM NaHCO_3_. This was followed by incubation in Neuronal Isolation Enzyme with Papain (200 μl per 10 aggregates) for 20–30 min at 37 °C, with periodic agitation. After careful removal of the enzyme solution, aggregates were washed gently three times with HHGN. Dissociation to a single cell suspension was performed by trituration 20–25 times in 500 μl seeding medium, consisting of DMEM/F12 with L-glutamine, supplemented with N2 (1%), 1.4 mM additional L-glutamine, 5 μg/ml additional insulin (Sigma-Aldrich), penicillin/streptomycin (1%) and 10% HyClone US-defined heat-inactivated foetal bovine serum (FBS, GE Healthcare Life Sciences, Little Chalfont, UK), taking care to avoid air bubble formation. Cells were then pooled, and seeding medium added to 5 ml, before centrifugation at 185*×g* (900 rpm) for 5 min. Following centrifugation, the supernatant was removed and the pellet resuspended in 250 μl seeding medium. Cells were counted, and the concentration adjusted to 8 × 10^6^ cells/ml in seeding medium.

In parallel, mouse cerebellar cells were prepared. Pregnant dams (C57BL/6) at 18 days of gestation were sacrificed, and cerebella were dissected from the pups. Approximately one litter was used for each iPSC line. Cerebella were washed twice with HHGN, and incubated in TrypLE Express for 10 min at 37 °C, with periodic agitation. This was followed by a further three washes with HHGN, before dissociation by trituration in seeding medium, centrifugation and resuspension at 8 × 10^6^ cells/ml, as described above.

Human and mouse cells were mixed at a ratio of 1:10, and 55 μl of the mixed cell suspension was seeded as a small bubble on Cell Desk LF plastic coverslips (Sumitomo Bakelite), previously coated with poly-L-ornithine (0.5 mg/ml, Sigma-Aldrich) and natural mouse laminin (10 mg/ml). After incubation for 3 h at 37 °C, 5% CO_2_, 500 μl of culture medium, consisting of DMEM/F12 with L-glutamine, supplemented with N2 (1%), 1.4 mM additional L-glutamine, 5 μg/ml additional insulin (Sigma-Aldrich), penicillin/streptomycin (1), 0.5 ng/ml tri-iodothyronine (T3, Sigma-Aldrich), 100 μg/ml BSA (Sigma-Aldrich), 50 ng/ml recombinant human brain-derived neurotrophic factor (BDNF, R&D Systems) and 50 ng/ml recombinant human neurotrophin 3 (NT3, R&D Systems), was gently added to each well to reduce the final serum concentration. A half-volume replacement was performed once a week, using fresh feeding medium, consisting of culture medium, additionally supplemented with 4 μM of the glial proliferation inhibitor cytosine β-D-arabinofuranoside (AraC, Sigma-Aldrich), and 5-10 μg/ml laminin.

### Immunocytochemistry

Cell aggregates were harvested for immunocytochemistry at days 21 and 35 of differentiation. In both cases, aggregates were fixed for 15–20 min at 4 °C in 4% paraformaldehyde, before processing for cryosectioning [17]. Ten-micrometre cryosections were subjected to immunocytochemistry as previously described [12].

For mixed co-cultures, immunocytochemistry was performed at days 50, 70 and 90, as follows: cells were fixed for 20 min at room temperature (RT) in prewarmed 4% paraformaldehyde, before washing in PBS, and permeabilisation for 20 min at RT in 0.4% (*v*/*v*) Triton X-100 in PBS. This was followed by blocking for 1 h at RT in 10% (*w*/*v*) skim milk powder and 1% (*v*/*v*) normal goat serum in TBST (150 mM NaCl, 10 mM Tris pH 8, 0.05% Tween 20). Following overnight incubation at 4 °C with primary antibody diluted in 3% (*w*/*v*) bovine serum albumin (BSA) in TBST, coverslips were washed three times in PBS and incubated for 2 h at RT with the relevant fluorescent-conjugated secondary antibody. Finally, coverslips were mounted onto slides in mounting medium containing DAPI (Vector Laboratories, Peterborough, UK). A list of primary and secondary antibodies can be found in Supplementary Table 1.

Analysis of cell morphology and differentiation was performed across three separate differentiation experiments, using at least 10 images per experiment. Results were quantified manually using ImageJ (http://imagej.nih.gov/ij).

### Quantitative Real-Time PCR

RNA was isolated from cell aggregates on days 21 and 35 using the RNeasy Micro Kit (Qiagen, Hilden, Germany) and reverse transcribed to cDNA using the SuperScript III First-Strand Synthesis System. Quantitative real-time PCR was performed using the Fast SYBR Green Master Mix (Applied Biosystems, Foster City, CA, USA) and gene-specific primers (Supplementary Table 2), on the Applied Biosystems StepOne Plus qPCR machine, and results analysed using the ΔΔCt method, on the StepOne Software v2.0 (Applied Biosystems). Statistical analysis was performed on GraphPad Prism, using a two-tailed Student’s *t* test, assuming unequal variances.

## Results

### Generation of Cerebellar Progenitors in 3D Culture

**Fig. 2:**
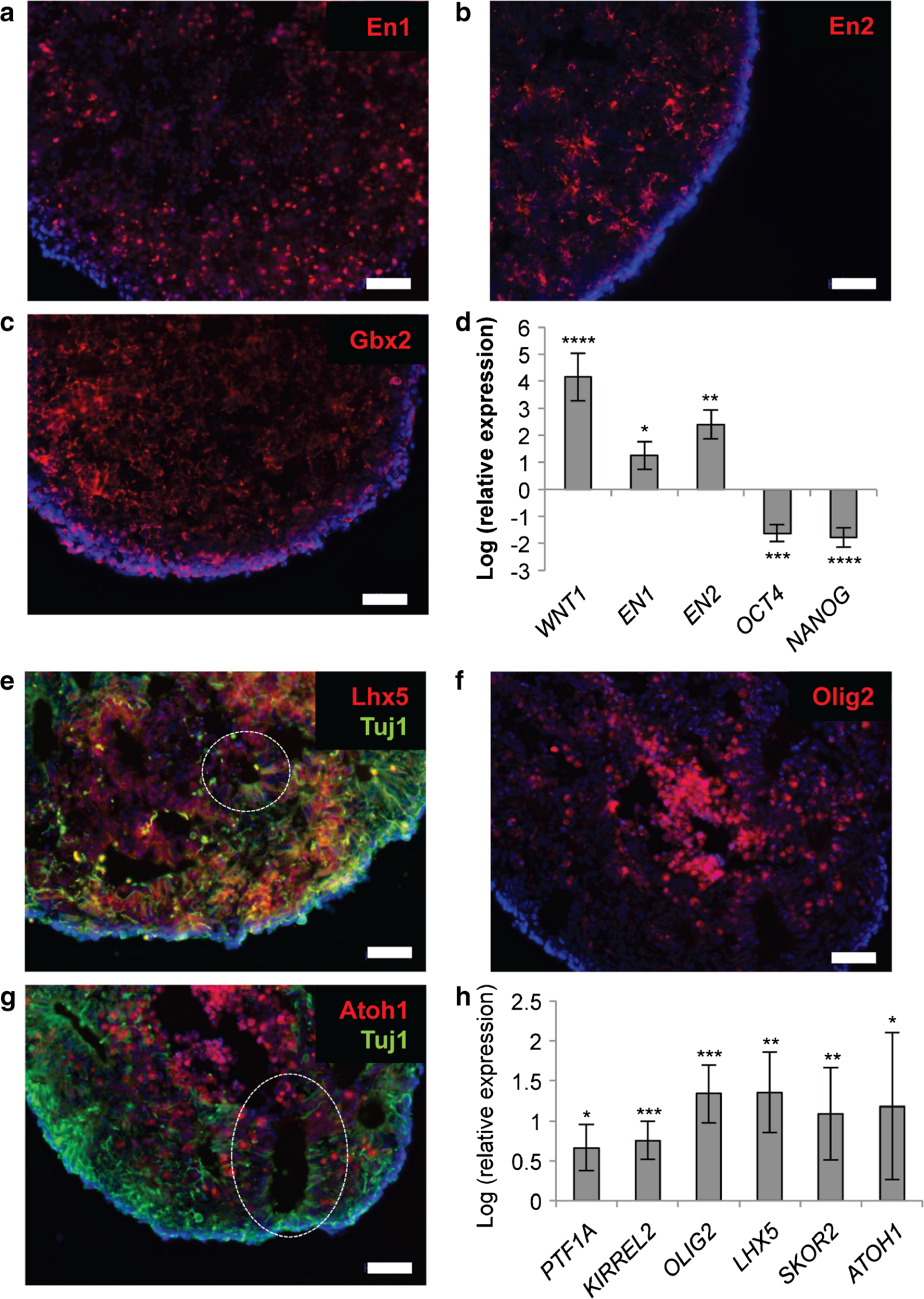
Generation of cerebellar progenitors from hiPSCs. After 21 days in suspension culture, hiPSC aggregates express hindbrain-specific transcription factors En1 (**a**), En2 (**b**) and Gbx2 (**c**), and the isthmic organiser factor *WNT1*, and show suppression of the pluripotency genes *OCT4* and *NANOG* (**d**). By day 35, subpopulations of cells within hiPSC aggregates express the early neuronal marker Tuj1 (βIII-tubulin), as well as Purkinje cell precursor markers Lhx5 (**e**), Olig2 (**f**) and the granule cell marker Atoh1 (**g**). Expression of the ventricular zone (GABAergic) marker *PTF1A*, and the rhombic lip (glutamatergic) marker *ATOH1*, as well as additional Purkinje cell markers *KIRREL2* and *SKOR2*, was confirmed by qPCR (**h**). Nuclei are stained with DAPI (blue). Examples of neural rosettes are indicated by a dashed line. Scale bar 50 μm. Gene expression is shown relative to hiPSCs (set to zero), normalised to β-actin. Results are representative of five biological replicates, except in the case of *PTF1A*, where n=2; error bars represent SD. *p < 0.05; **p < 0.01; ***p < 0.001; ****p < 0.0001

Using a combination of three factors selected to mimic the self-inductive properties of the isthmic organiser—insulin, fibroblast growth factor 2 (FGF2) and the transforming growth factor β (TGFβ)-receptor blocker SB431542—we demonstrated robust differentiation of human iPSCs (hiPSCs) into cerebellar progenitors. Following dissociation, reaggregation in low-adhesion V-bottomed 96-well plates and culture in suspension for 14 to 21 days, the hiPSC aggregates showed reproducible upregulation of the early midbrain-hindbrain markers *EN1*, *EN2*, *GBX2* and *WNT1*, and suppression of the pluripotency genes *NANOG* and *OCT4* (Fig. 2a–d). Seventy to 90% of cells in aggregates were positive for *EN1* by immunostaining at this point (average across 15 separate experiments, involving three different iPSC lines).

Of note, several modifications from existing differentiation protocols were found to dramatically enhance survival and promote reaggregation of hiPSCs within the first 24 h after dissociation. These included the addition of the ROCK inhibitor Y-27632 at a concentration of 50 μM (2.5–5 times the concentration previously reported [13, 18]), and adjustment of the seeding density of cells from 6000/well [13] to 12,000/well [18]. Furthermore, we observed a reduction in concatenation of hiPSC aggregates, associated with easier handling and improved longer term growth, when cells were cultured in non-adherent 24-well plates from days 21–35.

As early as day 35 of differentiation, subpopulations of cells within hiPSC aggregates were found to express markers of the two cerebellar germinal zones: the ventricular zone marker *PTF1A* and the rhombic lip marker *ATOH1*, indicating the presence of GABAergic and glutamatergic precursors, respectively (Fig. 2e–h). GABAergic precursors were further found to express Purkinje cell progenitor markers, including Lhx5, Olig2, Kirrel2 and Skor2 (Fig. 2e–h). Rosette-like structures resembling neuroepithelium were also frequently observed at this stage (Fig. 2e–g).

### Differentiation of Purkinje Cells from Cerebellar Progenitors

On day 35 of differentiation, hiPSC aggregates were dissociated into a single-cell suspension of cerebellar progenitors and co-cultured with cerebellar cells derived from E18.5 mouse embryos in medium containing BDNF and NT3, in order to promote maturation of human Purkinje cells (Fig. 1). In contrast to existing protocols [13, 18], we did not perform fluorescent-activated cell sorting for Kirrel2-positive human cells. Additional modifications included the use of dissociated whole cerebella from E18.5 mouse pups, rather than E14.5 rhombic lip cultures [13, 18] in order to enhance the number of cells available for co-culture, and the addition of 5–10 μg/ml of laminin to the feeding medium from day 42, to provide additional structural support to maturing cultures.

**Fig. 3:**
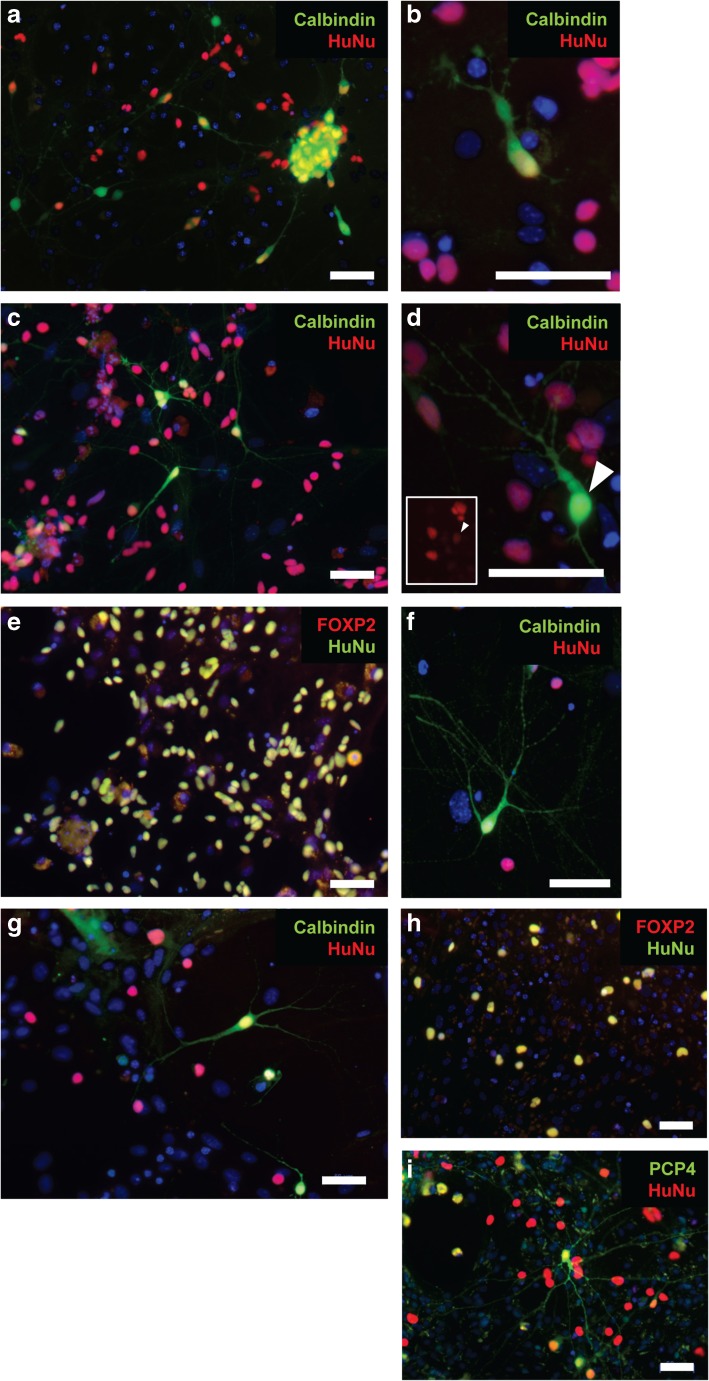
Differentiation of Purkinje-like cells from cerebellar progenitors. Immunostaining of dissociated neuronal cultures at day 50 shows human Purkinje cell precursors positive for the specific marker calbindin, which have begun to develop a rudimentary dendritic arbour (**a**, **b**). By day 70, cells continue to mature, developing more complex dendritic arbours, and staining positive for calbindin (**c**, **d**) and Foxp2 (**e**). At this stage, human cells make up 42.7 ± 23.5% of the total cell population, with approximately 10% staining positive for calbindin. After a further 20 days in culture, dendritic arbours continue to develop. HuNu-positive cells make up 30.2 ± 28.5% of the culture at day 90, with approximately 11.9% of these staining positive for calbindin (**f**, **g**). Foxp2 (**h**) and Pcp4 (**i**) expression could also be detected at this stage, suggesting continued maturation of these cells in culture. Nuclei are stained with DAPI (blue). Scale bar 50 μm

Purkinje cell precursors which showed evidence of a rudimentary dendritic arbour, and which stained positive for both human nuclear antigen (HuNu) and the later Purkinje cell marker calbindin, were observed as early as day 15 of co-culture (day 50 of differentiation) (Fig. 3a, b). By day 70 of differentiation, these cells had developed more complex dendritic arbours (2.8 ± 0.4 dendrites per cell, with a maximum degree of branching of 2.8 ± 0.6 branches per dendrite). HuNu-positive cells made up 42.7 ± 23.5% of the total cell population, with approximately 10 ± 5.7% of these cells staining positive for calbindin (Fig. 3b). After a further 20 days of differentiation, the number of HuNu-positive cells had declined slightly (30.2 ± 28.5%), although the proportion of these which stained positive for calbindin remained constant (11.9 ± 0.5%). Dendritic arbours continued to develop (3.6 ± 0.0 dendrites per cell, with a maximum degree of branching of 3.1 ± 0.1 branches per dendrite) (Fig. 3c, d).

Expression of additional Purkinje cell makers, Pcp4 and Foxp2, was also observed at these later stages of differentiation, suggesting further specification of these cells towards the correct lineage (Fig. 3e, h, i).

It should be noted that this protocol supports the growth of mouse Purkinje cells (which stain positive for calbindin, but not HuNu) for approximately 2 weeks. However, these cells represented a much smaller proportion of the total than human Purkinje cells, particularly when co-culture was performed using cerebella obtained from E18.5 mouse pups, and they do not persist beyond day 50 of the differentiation.

## Discussion

We have developed a simplified protocol for the reproducible generation of cerebellar cells from hiPSCs in vitro, which successfully recapitulates early cerebellar development, as assessed by comparison with gene expression in the embryonic mouse brain.

Treatment of aggregated hiPSCs with insulin and the caudalising factor FGF2 resulted in dramatic induction of endogenous isthmic organiser factor genes, including *WNT1* and *GBX2*, reminiscent of gene expression patterns typically observed at the midbrain-hindbrain boundary [4, 19]. By 35 days in culture, these hiPSC aggregates had begun to differentiate into cells representative of the two cerebellar germinal layers, suggesting the presence of both GABAergic and glutamatergic progenitors, which could, in theory, both be further expanded and matured [5].

For our purposes, we focused on the further maturation of Purkinje cells, demonstrating the presence of human calbindin-positive cells after 15 days of co-culture with mouse cerebellar progenitors. In the presence of BDNF and NT3 (known to promote survival and phenotypic differentiation of Purkinje cells [20, 21]), these calbindin-positive cells could be maintained in culture for at least 55 days, during which time they continued to undergo lineage specification, producing increasingly elaborate dendritic arbours, and expressing additional markers of Purkinje cell development, including FOXP2 and PCP4. Future work will include immunostaining for markers of mature Purkinje cells, as well as detailed electrophysiology, in order to assess the continuing maturity of these cells in long-term culture.

The establishment of a robust protocol for generating Purkinje cells from hiPSCs is of particular importance, given their central role in cerebellar function, and hence, in the development of diseases such as the cerebellar ataxias [6]. To date, however, the very properties which make Purkinje cells so vulnerable in disease (including their size, morphological complexity and electrophysiological properties) have made them difficult to differentiate and culture in vitro, with existing protocols proving lengthy and challenging to reproduce [8]. Indeed, only one study has thus far succeeded in generating cerebellar Purkinje cells from a patient with cerebellar disease (spinocerebellar ataxia type 6) [18].

In an attempt to overcome these challenges, our simplified differentiation approach incorporated several key modifications to existing protocols. Many of these were designed to enhance cell survival and maturation, including the addition of the apoptosis inhibitor Y-27632 at increased concentrations, and incorporation of laminin into the co-culture feeding medium, to enhance cellular adhesion and provide additional structural support to fragile neuronal processes during maturation. Cells were also seeded at a higher density at both day 0 and day 35, in order to maintain vital cell-cell contacts during both stages of dissociation and replating. Additional modifications were adopted to improve the ease of handling without compromising the quality of cells generated, including the use of smaller format dishes for suspension culture between days 21 and 35, and the use of E18.5 cerebellar cultures, rather than the technically challenging preparation and lower cell yield of E14.5 rhombic lip cultures, as a source of cells for co-culture. By following these adaptations, we have consistently generated cultures containing approximately 10% Purkinje cells. However, given the widespread expression of FOXP2, it may also be possible to further improve the yield of Purkinje cells in culture, through modifications to the media composition or culture conditions. Using this new protocol, we hope to make the differentiation of cerebellar neurons from hiPSCs more tractable for future studies.

Despite the promise of hiPSC-derived models of cerebellar development and disease, a number of challenges remain to be addressed. While two-dimensional neuronal cultures facilitate investigations into the development of individual cells, they face significant limitations in terms of long-term survival and maturation, as a result of a lack of structural and trophic support. Additionally, phenotypes arising from cell-environment interactions or non-cell autonomous mechanisms, such as those affecting neuronal migration and synapse formation, may be lost in these cultures. It may therefore become necessary in the future to supplement results from these models with investigations using three-dimensional organoid cultures, which deliberately include multiple cell types, and mimic the in vivo architecture of a particular brain region [22].

Concerns have also been raised regarding the correlation between iPSC-derived models and neurons in the human brain, particularly in regard to age and disease stage. Despite detection of expression of mature neuronal markers in some models, transcriptomic analyses suggest that iPSC-derived neurons are generally still in an embryonic state [23], as is true of the cerebellar neurons described here. Thus, while these cells are likely to be extremely useful for the study of neurodevelopmental defects and early stages of disease progression, it is likely that further functional maturation, and/or the use of exogenous stressors, will be necessary in order to fully recapitulate the phenotypes of late-onset neurodegenerative disease [24–26].

The use of mouse cerebellar progenitors represents one approach to enhance maturation and provide vital trophic support to developing human Purkinje cells in vitro [13]. While simple mixed co-culture approaches are extremely useful for studies of morphology and maturation through imaging, larger scale biochemical analyses will require the removal of confounding mouse cells, for example, through the use of embryos from transgenic reporter mice, enabling purification of mouse and human cultures by FACS prior to analysis.

## Conclusion

iPSC-based models offer distinct advantages in the study of cerebellar development and neurodegeneration, enabling investigations into disease mechanisms and therapeutic development using disease-relevant patient cells. Past approaches to differentiate cerebellar neurons from hiPSCs have proven lengthy, technically challenging and difficult to reproduce, and it is our hope that continued innovations, such as the simplified protocol described here, may make these models more accessible, in order to enable future advances in the field.

## Electronic Supplementary Material

ESM 1 (DOCX 1.04 MB)
