# Supplementary material for: A Simplified Method for Generating Purkinje Cells from Human-Induced Pluripotent Stem Cells
Source: Cerebellum. 2018 Feb 3;17(4):419–27. doi: 10.1007/s12311-017-0913-2 (PMC6028833; doi:10.1007/s12311-017-0913-2)
Supplement: Supplementary file 1 — (DOCX 1.04 MB) [file 12311_2017_913_MOESM1_ESM.docx]

**A simplified method for generating Purkinje cells from human induced pluripotent stem cells**

Lauren M Watson^1*^, Maggie MK Wong^1^, Jane Vowles^2^, Sally A Cowley^2^, Esther BE Becker^1^

^1^Department of Physiology, Anatomy and Genetics, University of Oxford, Oxford, United Kingdom

^2^Sir William Dunn School of Pathology, University of Oxford, Oxford, United Kingdom

*To whom correspondence should be addressed: [lauren.watson@dpag.ox.ac.uk](mailto:lauren.watson@dpag.ox.ac.uk)

**Supplementary Information**

**Supplementary Methods**

*Reprogramming donor fibroblasts to iPSC*

Reagents were obtained from Invitrogen, unless stated otherwise. iPSCs were derived from skin biopsy fibroblasts in the James Martin Stem Cell Facility, University of Oxford under standardised protocols, using the SeVdp(KOSM)302L Sendai virus system [1–3], containing the genes for all four reprogramming transcription factors (*KLF4, OCT4, SOX2, C-MYC*) expressed from a single transcript, packaged into a single Sendai virus, ensuring gene dosage ratio consistency. The system contains a target for mir302, ensuring viral RNA is efficiently degraded once cells are reprogrammed to pluripotency (when mir302 is expressed), hence complete removal of exogenous genetic material within a few passages. Briefly, transduced fibroblasts were cultured on mitotically inactivated mouse embryonic feeder cells (MEFs; derived from outbred Swiss mice, Department of Pathology, Oxford [4,5], or CF1 MEFs (Merck Millipore, Billerica, MA, USA) on 0.1% gelatin coated plates, cultured in knock-out Dulbecco's modified Eagle's medium (DMEM), 10% knock-out-serum replacement, 2mM Glutamax-I, 100U/mL penicillin, 100µg/mL streptomycin, 1% nonessential amino acids, 0.5mM β-mercaptoethanol, 10ng/mL basic fibroblast growth factor (bFGF) (R&D Systems). Colonies picked on day 21–28 were passaged manually every 5–7 days, and then adapted to feeder-free conditions on Matrigel-coated plates (Corning hESC-qualified matrigel) in mTeSR1 (Stem Cell Technologies), passaged with 0.5mM EDTA, generating quality-controlled, cryopreserved master-stocks (p10-20, by which time the virus has automatically cleared). iPSC lines tested negative for mycoplasma using MycoAlert (Lonza, Basel, Switzerland). Genome integrity analysis was performed used Illumina Human CytoSNP-12v2.1 beadchip array (300,000 markers), and KaryoStudio and GenomeStudio software (Illumina, San Diego, CA, USA). iPSC line SNP profiles were compared to the original pool of fibroblasts, which confirmed the identity of the iPSC to the original fibroblasts. To assess conformity to pluripotent profiles, RNA was extracted from iPSC using an RNeasy kit (Qiagen, Hilden, Germany) for Illumina HT12v4 transcriptome array analysis and image data files uploaded to www.pluritest.org were scored for pluripotency [6]. iPSC lines were also assessed for pluripotency markers by flow cytometry. Cells lifted with TrypLE, were fixed (4% paraformaldehyde, PFA, in phosphate-buffered saline, PBS, 10 min), permeabilized (−20°C methanol), washed and stained in flow cytometry buffer (PBS, 10 µg/mL human IgG (Sigma), 1% fetal calf serum (Hyclone) and 0.01% sodium azide) with antibody or isotype-matched control (same fluorophore/manufacturer, 1µg antibody per million cells) for 30 minutes, washed, measured using a FACS Calibur (Becton Dickinson, Oxford, UK), and analyzed using FlowJo software. Antibodies (clone, isotype control, supplier): TRA-1-60 (B119983, IgM-488, Biolegend, San Diego, CA, USA), NANOG (D73G4, IgG-647, Cell Signaling Technology, Danvers, MA, USA).

**
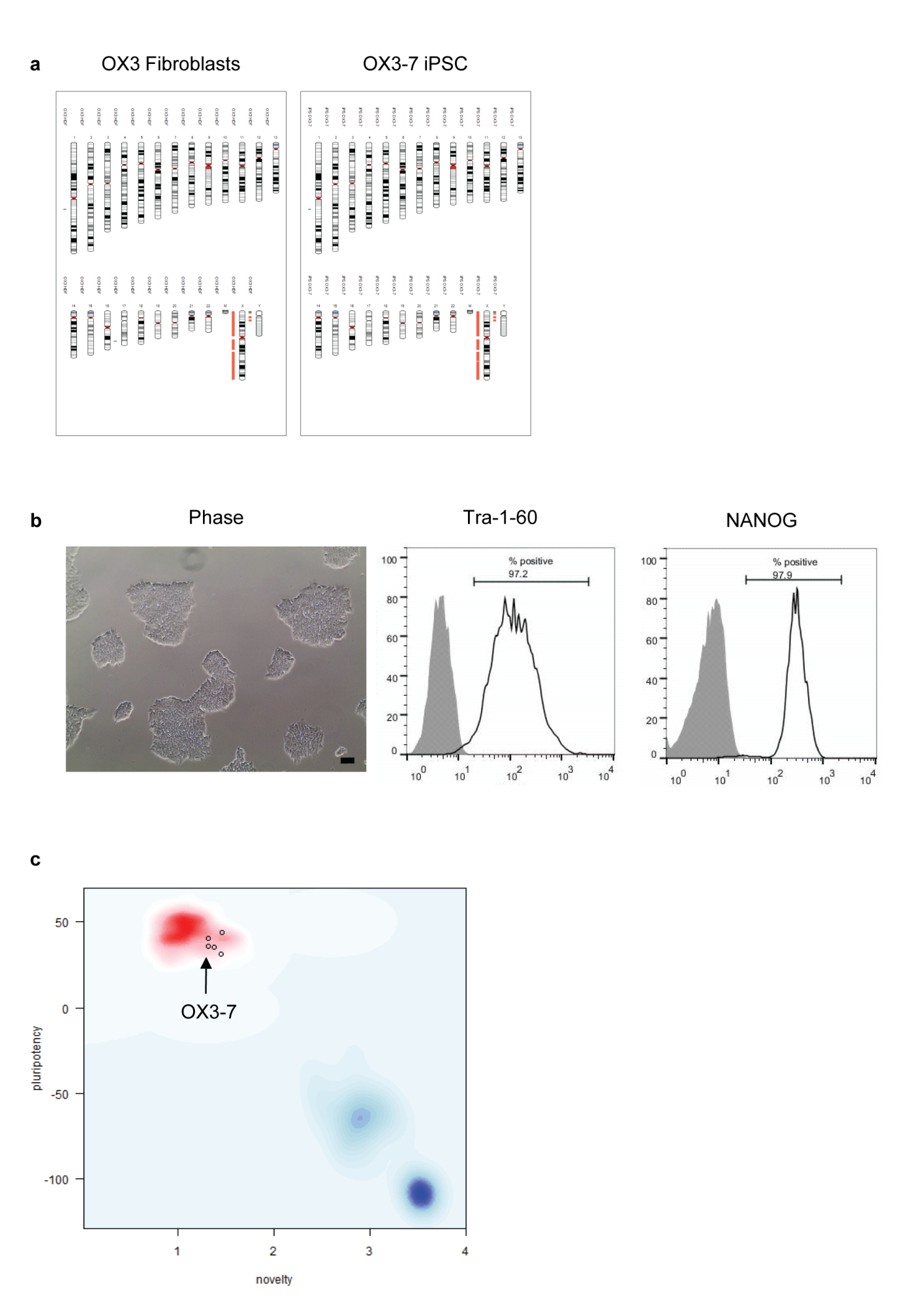
**

**Fig. S1.** Characterisation of previously unpublished iPSC line used in this study. **a** Karyostudio Karyograms of iPSC OX3-7 versus the original OX3 fibroblasts. Annotation colours: Autosomal detected regions deviating from reference datasets, green (amplification), orange (deletion), grey (loss of heterozygosity); X chromosome grey (two-copy, female), orange (single copy, male). **b** Left panel, pluripotent stem cell-like morphology of iPSC OX3-7 (cell-cell-contact-dependent clusters, with high nucleus to cytoplasm ratio) by phase microscopy in feeder-free culture; Scale bar 100 µm; Right panel, FACS analysis of iPSC for pluripotency markers Tra-1-60 and Nanog (black line); grey filled plot, isotype control. **c** PluriTest analysis of Illumina HT12v4 transcriptome array data shows that the iPSC OX3-7 clusters with pluripotent stem cells (top left quadrant), not with partly- or differentiated cells (bottom right quadrant); *y* axis pluripotency score, *x* axis novelty score.

**Supplementary Table 1. Primary and secondary antibodies**

| **Antibody** | **Company** | **Host Species** | **Dilution** |
| --- | --- | --- | --- |
| **Primary Antibodies** | | | |
| Atoh1 | EMD Millipore | Rabbit | 1:200 |
| Calbindin | Swant | Rabbit | 1:5000 |
| En1 | Abcam | Rabbit | 1:50 |
| En2 | Santa Cruz | Goat | 1:50 |
| Foxp2 | Abcam | Goat | 1:5000 |
| Gbx2 | Santa Cruz | Goat | 1:50 |
| Human Nuclear Antigen | Abcam | Mouse | 1:200 |
| Lhx5 | EMD Millipore | Rabbit | 1:100 |
| Olig2 | R&D Systems | Goat | 1:40 |
| Pcp4 | Santa Cruz | Rabbit | 1:1000 |
| Tuj1 | Covance | Mouse | 1:1000 |
| **Secondary Antibodies** | | | |
| Anti-Goat Alexa 594 | Invitrogen | Donkey | 1:1000 |
| anti-Mouse Alexa 488 | Invitrogen | Goat | 1:1000 |
| anti-Mouse Alexa 594 | Invitrogen | Goat | 1:1000 |
| anti-Rabbit Alexa 488 | Invitrogen | Goat | 1:1000 |
| anti-Rabbit Alexa 594 | Invitrogen | Goat | 1:1000 |

**Supplementary Table 2. Primers for qPCR**

| **Gene** | **Forward Primer (5’-3’)** | **Reverse Primer (5’-3’)** | **Reference** |
| --- | --- | --- | --- |
| *ATOH1* | TGTTATCCCGTCGTTCAACAAC | TGGGCGTTTGTAGCAGCTC | [7] |
| *EN1* | GCTTGTCCTCCTTCTCGTTC | TGGTCAAAACTGACTCGCAG | - |
| *EN2* | CCGGCGTGGGTCTACTGTA | GGCCGCTTGTCCTCTTTGTT | [7] |
| *KIRREL2* | GGGGCTAGTTCAGTGGACTAA | CACGGGCCTAATGTGGAGG | [7] |
| *LHX5* | GTGCGCGAAGAAGTCGTAGT | CGAGTCTGAGATGTTGGGGT | - |
| *NANOG* | CAGCCCCGATTCTTCCACCAG | CGGAAGATTCCCAGTCGGGTT | - |
| *OCT4* | GACAGGGGGAGGGGAGGAGC | CTTCCCTCCAACCAGTTGCCC | - |
| *OLIG2* | GATAGTCGTCGCAGCTTTCG | CCTGAGGCTTTTCGGAGC | - |
| *PTF1A* | TGAGTTGTTTTTCATCAGTCCA | CAGGCCCAGAAGGTCATC | - |
| *SKOR2* | AGCCCAGTTCACCATCCAT | GCTGTTGTCATCCTTTGTAGATAC | - |
| *WNT1* | CAACCGAGGCTGTCGAGAAA | GTGCAGGATTCGATGGAACCT | [7] |
| *Β-ACTIN* | GCCGCCAGCTCACCATGGATG | CCATCACGCCCTGGTGCCTGG | - |

**References**

1. Nishimura K, Sano M, Ohtaka M, Furuta B, Umemura Y, Nakajima Y, et al. Development of defective and persistent Sendai virus vector: A unique gene delivery/expression system ideal for cell reprogramming. J Biol Chem. 2011;286:4760–71.

2. Nishimura T, Kaneko S, Kawana-Tachikawa A, Tajima Y, Goto H, Zhu D, et al. Generation of rejuvenated antigen-specific T cells by reprogramming to pluripotency and redifferentiation. Cell Stem Cell. 2013;12:114–26.

3. Dafinca R, Scaber J, Ababneh N, Lalic T, Weir G, Christian H, et al. C9orf72 Hexanucleotide expansions are associated with altered endoplasmic reticulum calcium homeostasis and stress granule formation in induced pluripotent stem cell-derived neurons from patients with amyotrophic lateral sclerosis. Stem Cells. 2016;34:2063–78.

4. Chia R, Achilli F, Festing MF, Fisher EM. The origins and uses of mouse outbred stocks. Nat Genet. 2005;37:1181–6.

5. Gardner RL. Investigation of cell lineage and differentiation in the extraembryonic endoderm of the mouse embryo. J Embryol Exp Morphol. 1982;68:175–98.

6. Müller F, Schuldt BM, Williams R, Mason D, Altun G, Papapetrou E, et al. A bioinformatic assay for pluripotency in human cells. Nat Methods. 2011;8:315–7.

7. Muguruma K, Nishiyama A, Kawakami H, Hashimoto K, Sasai Y. Self-organization of polarized cerebellar tissue in 3D culture of human pluripotent stem cells. Cell Rep. 2015;10:537–50.
